# 3D Bioprinted Scaffolds for Bone Tissue Engineering: State-Of-The-Art and Emerging Technologies

**DOI:** 10.3389/fbioe.2022.824156

**Published:** 2022-04-11

**Authors:** Zahra Yazdanpanah, James D. Johnston, David M. L. Cooper, Xiongbiao Chen

**Affiliations:** ^1^ Division of Biomedical Engineering, College of Engineering, University of Saskatchewan, Saskatoon, SK, Canada; ^2^ Department of Mechanical Engineering, College of Engineering, University of Saskatchewan, Saskatoon, SK, Canada; ^3^ Department of Anatomy Physiology and Pharmacology, College of Medicine, University of Saskatchewan, Saskatoon, SK, Canada

**Keywords:** bioprinting, 3D printing, bone tissue engineering, scaffold, critical-sized defect

## Abstract

Treating large bone defects, known as critical-sized defects (CSDs), is challenging because they are not spontaneously healed by the patient’s body. Due to the limitations associated with conventional bone grafts, bone tissue engineering (BTE), based on three-dimensional (3D) bioprinted scaffolds, has emerged as a promising approach for bone reconstitution and treatment. Bioprinting technology allows for incorporation of living cells and/or growth factors into scaffolds aiming to mimic the structure and properties of the native bone. To date, a wide range of biomaterials (either natural or synthetic polymers), as well as various cells and growth factors, have been explored for use in scaffold bioprinting. However, a key challenge that remains is the fabrication of scaffolds that meet structure, mechanical, and osteoconductive requirements of native bone and support vascularization. In this review, we briefly present the latest developments and discoveries of CSD treatment by means of bioprinted scaffolds, with a focus on the biomaterials, cells, and growth factors for formulating bioinks and their bioprinting techniques. Promising state-of-the-art pathways or strategies recently developed for bioprinting bone scaffolds are highlighted, including the incorporation of bioactive ceramics to create composite scaffolds, the use of advanced bioprinting technologies (*e.g.*, core/shell bioprinting) to form hybrid scaffolds or systems, as well as the rigorous design of scaffolds by taking into account of the influence of such parameters as scaffold pore geometry and porosity. We also review *in-vitro* assays and *in-vivo* models to track bone regeneration, followed by a discussion of current limitations associated with 3D bioprinting technologies for BTE. We conclude this review with emerging approaches in this field, including the development of gradient scaffolds, four-dimensional (4D) printing technology via smart materials, organoids, and cell aggregates/spheroids along with future avenues for related BTE.

## Introduction

Bone is a resilient tissue with self-healing capacity. However, a large bone defect, referred to as a critical-sized defect (CSD), cannot be healed by the patient’s body (Schroeder and Mosheiff, 2011; Park et al., 2015). The size of CSDs can vary by the skeletal region involved and the state of soft tissue surrounding it (Nauth et al., 2011; Schemitsch, 2017). For example, a 3-cm diameter defect is regarded as a CSD for the radius and ulna, while a 5-cm diameter defect is classified as a CSD for the femur and tibia (Calori et al., 2011; Stewart et al., 2015). In these cases, surgical interventions such as bone grafts are needed to restore the function of the bone (Ashammakhi et al., 2019). Traditional therapeutic approaches such as autografts, allografts, and xenografts have been restricted due to associated drawbacks such as limited donor supply and donor sites, additional surgery, the potential risk of disease transmission, and immune response after implantation. Bone tissue engineering (BTE) has drawn significant attention to the creation of novel constructs to restore, maintain and/or improve bone function (Guarino et al., 2007; Wang and Yeung, 2017; Turnbull et al., 2018).

Scaffolds, cells, and cytokines are key components in BTE. Scaffolds are 3D structures providing a temporary environment for extracellular matrix (ECM) formation and cellular activity as well as diffusion of oxygen, nutrient delivery, and waste removal. These 3D structures must also provide mechanical support to resist external forces and gradually remodel over time as new bone tissue is formed (Seol et al., 2013).

Conventional fabrication methods such as solvent-casting (Kim et al., 2008; Aboudzadeh et al., 2010), particulate-leaching (Guarino et al., 2008; Verma et al., 2019), and freeze drying (Zhang and Ma, 1999; Niu et al., 2009; Aboudzadeh et al., 2010; Zhu et al., 2011; Cholas et al., 2016) have had limited capacity to control pore size, pore geometry, pore interconnectivity, and the spatial distribution of pores in scaffolds. Conversely, 3D printing, an advanced fabrication technique, is considered the most promising technique for creating biomedical scaffolds, artificial tissues, and organs due to its enhanced ability to control scaffold structure (Gu et al., 2016; Chen, 2018). Recent developments in 3D printing technology have also enabled the incorporation of living cells and growth factors into scaffolds during the fabrication process, an approach known as bioprinting, which subsequently creates biomimetic tissue (Bose et al., 2013; Zhu and Chen, 2013; Gu et al., 2016).

Here, we first provide a summary of concepts about bone structure and bone defects. We next consider different types of biomaterials, cells, and growth factors commonly used in bioprinting for BTE. We also review bioprinting techniques used in BTE, printability, as well as mechanical and osteoconductive properties of 3D printed bone scaffolds. In addition, we discuss *in-vitro* assays and *in-vivo* models to track bone regeneration using bone scaffolds. We then conclude with challenges in current studies and recommendations for future research.

## Bone Structure and Defects

Living bone is a heterogeneous composite material consisting mineral, collagen (type I), and water (Guarino et al., 2007; Wu et al., 2014). Moreover, there are small quantities of other organic materials such as polysaccharides, proteins, proteoglycans, sialoproteins, and lipids in this dynamic/vascularized tissue. Hydroxyapatite (HAp), which is the major component of bone mineral, is responsible for proper provision of structural support (Wu et al., 2014; Yazdanpanah et al., 2015). In addition, bone has a cellular phase made of four main types of cells including osteoblasts (form bone tissue), osteoclasts (resorb bone tissue), osteocytes (maintain bone tissue), and bone lining cells (Hadjidakis and Androulakis, 2006; Guarino et al., 2007; Florencio-Silva et al., 2015).

**FIGURE 1:**
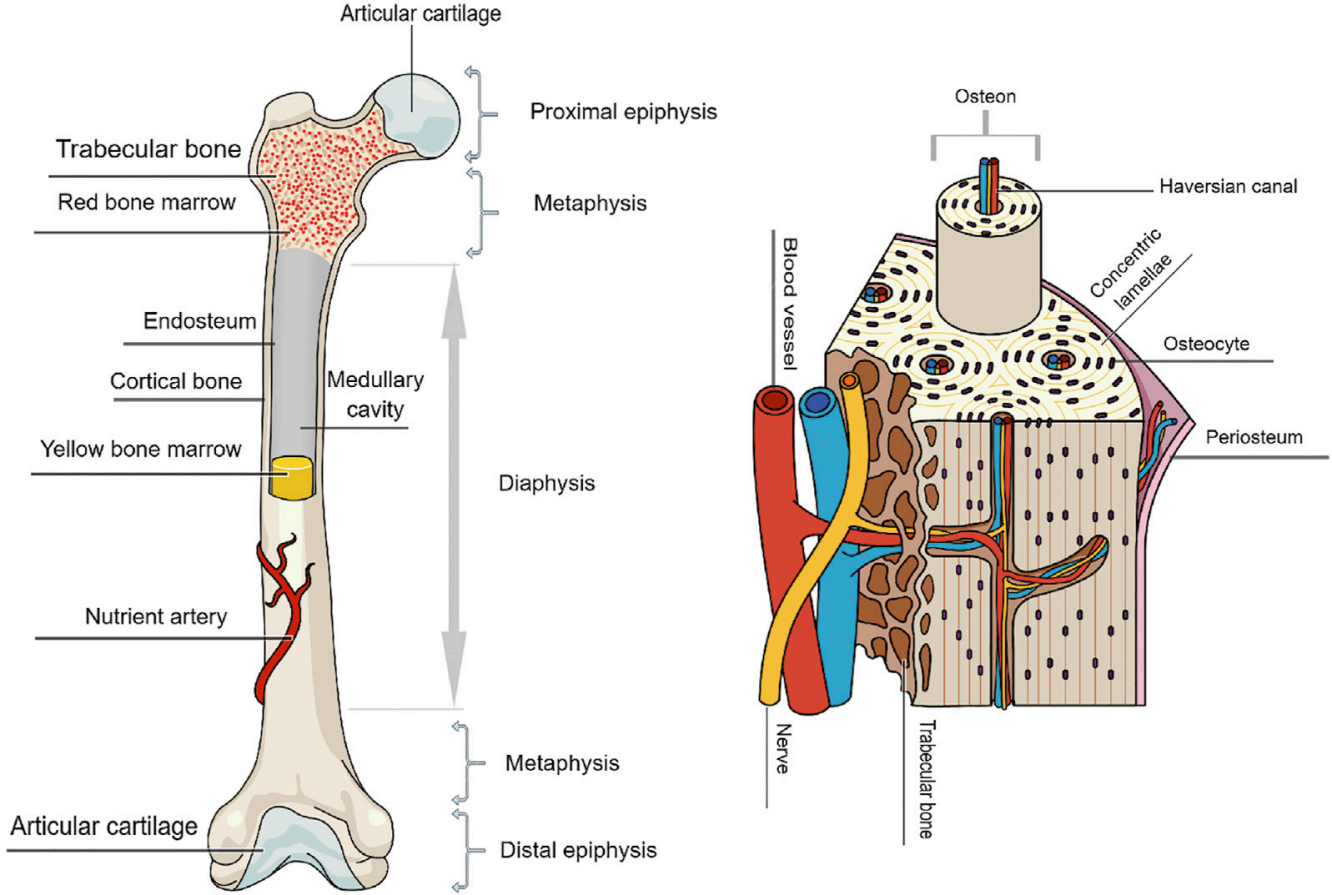
An overall schematic representation of long bone structure.

General categories of bones include long bones (*e.g.*, femur and tibia), short bones (*e.g.*, carpal and tarsal bones), flat bones (*e.g.*, skull), and irregular bones (*e.g.*, spinal elements). Long bones possess a hollow diaphysis, cone-shaped metaphyses, and rounded epiphyses (Clarke, 2008). Figure 1 illustrates the overall structure of long bone. At the macro-scale, a typical long bone includes cortical bone (compact bone), trabecular bone (cancellous or spongy bone), a periosteum, an endosteum, osteons (micro scale), collagen fibers (nanoscale), and collagen molecules (sub-nano scale). Cortical bone is dense, and is primarily responsible for providing mechanical support as well as protection. Conversely, trabecular bone, which has an open honeycomb-like structure, constitutes about 20% of skeleton mass and is typically located within the metaphyses/epiphyses at the ends of long-bones (Wu et al., 2014). Trabecular bone is less compact, more deformable, and, due to having a high surface area, more metabolically active than cortical bone (Hadjidakis and Androulakis, 2006). As it is a deformable structure, trabecular bone also plays a role in helping reduce dynamic forces associated with physiological loading (Downey and Siegel, 2006). Differences in mechanical properties between cortical and trabecular bone result from architectural differences, with cortical bone offering high resistance to axial, bending and torsional loading with a high compressive elastic modulus (E ≈ 7.0–30 GPa) (Hutmacher et al., 2007; Chatzistavrou et al., 2011) and compressive strength (S_c_ ≈ 100–230 MPa) (Hutmacher et al., 2007; Chatzistavrou et al., 2011) compared to that of trabecular bone (E ≈ 0.1–5 GPa, S_c_ ≈ 2–12 MPa) (Wu et al., 2014). The periosteum is a fibrous membrane of connective tissue observed on the bone surface, and the endosteum is a thin layer of lining cells found on the medullary cavity surface (Downey and Siegel, 2006; Wu et al., 2014). Osteons are vascular tunnels of cylindrical shape in which blood vessels and nerves are surrounded by concentric layers of bone called lamella (Guarino et al., 2007; Härle and Boudrieau, 2012; Olson and Carlson, 2017). What differentiates primary vs secondary osteons, also known as Haversian systems, is the way they are formed. Primary osteons are relatively small, less mineralized structures, formed in early life in locations where bone did not previously exist. During postnatal growth, resorption of existing bone occurs, and larger secondary osteons are deposited. These secondary osteons, which are the main structural unit of cortical bone, are constantly resorbed/renewed during life through the process of remodeling (Patterson-Kane and Firth, 2014). In trabecular bone, remodeling produces hemi-osteons, also known as trabecular packets, which have a similar layout to that of cortical bone osteon but are crescent-shaped (Parfitt, 1994; Dahl and Thompson, 2011; Patterson-Kane and Firth, 2014).

Bone is continuously renewed throughout life by remodeling, a cyclical process by which existing bone is replaced by new bone to maintain structural, biochemical, and biomechanical properties (Hadjidakis and Androulakis, 2006; Zhou et al., 2010). Remodeling, which is conducted by individual basic multicellular units (BMUs), is performed in three main stages, including: 1) bone resorption by osteoclasts; 2) reversal, which is a transition phase from resorption to formation; and 3) bone formation by osteoblasts (Hadjidakis and Androulakis, 2006; Kenkre and Bassett, 2018). Within the BMU, bone resorption is coupled with bone formation so that old bone is replaced by an equivalent amount of new bone to maintain skeletal balance (Martin and Rodan, 2008).

Bone defects can be caused by congenital abnormalities, trauma (*e.g.*, fractures and non-unions), bone disease (*e.g.*, osteoporosis, osteosarcoma, osteonecrosis), or surgery (*e.g.*, tumor removal, spinal fusion) (Gao et al., 2014; Loi et al., 2016; Ma et al., 2018; Ashammakhi et al., 2019) and have clinical as well as socioeconomic importance (Pneumaticos et al., 2010; Loi et al., 2016). Based on the American Academy of Orthopedic Surgeons (Stevens et al., 2008), 6.3 million fractures happen each year in the United States and half a million surgical procedures were performed in 2005 using autografts or allografts to repair bone defects, which cost approximately $2.5 billion USD. In 2011 (Loi et al., 2016), 465,070 spinal fusion treatments were performed using bone grafts in the United States. It has been reported that costs for treatment of bone fractures will reach $25 billion USD by 2025 (Loi et al., 2016).

Management of large bone defects, known as CSDs, is of great importance given they have negative effects on the patient’s quality of life due to prolonged hospitalizations and consecutive re-operations (Pneumaticos et al., 2010; Roseti et al., 2017). Despite the importance, no standard definition for a CSD has been reported in literature and the lack of consistency around its definition has led to conflicting opinions on their management (Schemitsch, 2017). General guidelines consider a defect size length greater than 1–2 cm and greater than 50% loss of the bone circumference as a CSD (Schemitsch, 2017). In addition, the classical definition of a CSD pertains to the smallest size intra-osseous wound in a particular bone and species of animal which is unable to heal during the lifetime of an animal (Pneumaticos et al., 2010; Spicer et al., 2012). Some others have also suggested that a defect is defined as a critical size when the length of deficiency is more than 2 or 3 times its diameter, or when a defect demonstrates less than 10% bone regeneration during the lifetime of the animal (Pneumaticos et al., 2010). The location and depth of the defect is also a consideration. Defects in the cortical diaphysis exhibit efficient regeneration of compact bone (Monfoulet et al., 2010). Defects though in distal and proximal epimetaphyses (which include both cortical and trabecular bone) exhibit efficient regeneration of trabecular bone but thin cortices, attributed to different bone-specific remodeling processes. The difference in healing process of various zones may come from the availability of endosteal, bone marrow, or bone lining cells (Monfoulet et al., 2010). A BTE study also found more bone formation in trabecular defect of a metaphyseal bone, which was implanted by bone scaffold with growth factor but a delayed cortical healing (Raina et al., 2019). To guide cortical regeneration, sealing the cortical defect endosteally with a collagen membrane loaded with growth factor was found promising (Raina et al., 2019).

### Bioink for Bioprinting

**FIGURE 2:**
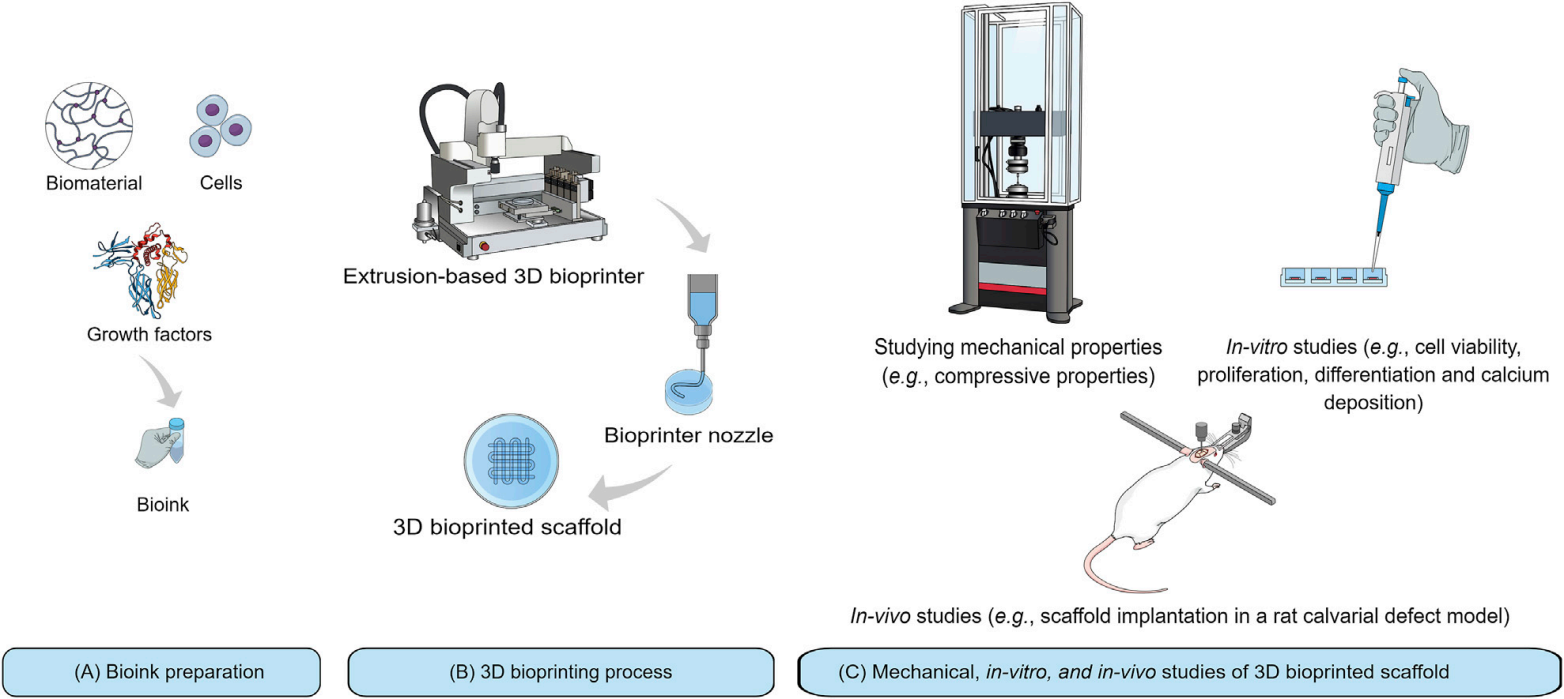
Schematic representation of 3D bioprinting technique and its application for BTE: **(A)** Bioink preparation from three components including biomaterial, growth factor, and cells; **(B)** Bioprinting process with an extrusion-based 3D bioprinter; and **(C)** Mechanical, *in-vitro*, and *in-vivo* studies.

Bioink (Figure 2A) is defined as a formulation of cells which may, but do not have to, contain biomaterials and biological components such as growth factors (Groll et al., 2018). A bioink should generally meet the biological and mechanical requirements of the bioprinting process. The biomaterial used in bioink must possess appropriate biocompatibility, bioactivity, and biodegradability (Salgado et al., 2004; Murphy and Atala, 2014; Mandrycky et al., 2016; Turnbull et al., 2018; Zimmerling et al., 2021). The biomaterial must also have proper viscosity for 3D printing process (Figure 2B), and have appropriate mechanical properties to provide sufficient load-carrying capacity as well as stiffness to maintain integrity of the bioprinted scaffold (Murphy and Atala, 2014; Munaz et al., 2016; Zhang et al., 2017). With regards to scaffold evaluations for BTE, it is necessary to study bioprinted scaffold in terms of mechanical properties followed by evaluating cell function and osteoconductive properties by *in-vitro* studies. Scaffold implantation into a bone defect in an animal model, *in-vivo* (Figure 2C), has also been a complementary approach to track the capability of a bone-like scaffold to regenerate new bone within a CSD.

#### Biomaterials

Given that biomaterial properties determine scaffold properties, biomaterial selection is a crucial step towards fabrication of bone-engineered constructs. To date, a wide variety of both natural and synthetic biomaterials have been used for producing bone scaffolds; however, not all provide a suitable matrix to embed cells (Salgado et al., 2004; Zhang et al., 2017). Accordingly, only a limited number of biomaterials are currently available for effective cell encapsulation.

In bioprinting, hydrogels are widely used as a matrix to encapsulate cells due to their biocompatibility, biodegradability, resemblance to the native ECM, and provision of a hydrated environment, which facilitate diffusion of nutrients, oxygen, and waste removal for cell growth (Huang et al., 2016; Bishop et al., 2017; Naghieh and Chen, 2021). A hydrogel for bioprinting must possess several characteristics (Du et al., 2015; Huang et al., 2016): 1) exhibit adequate rheological properties contributing to strands with good integrity, 2) maintain its shape during 3D printing and the pores should not collapse, and 3) non-toxic to cells and allow encapsulated cells to proliferate and differentiate.

Various natural hydrogels including alginate, gelatin, chitosan, collagen, hyaluronic acid (HA) have been commonly utilized for bioprinting (Huang et al., 2016). A summary of benefits and potential limitations of the above-mentioned natural hydrogels, specifically for the purposes of bioprinting bone scaffolds, is given in Table 1. Among natural hydrogels, alginate has been most commonly used in bioprinting of bone scaffolds due to its controllable degradation rate and the useful property of undergoing crosslinking, which allows it to be formulated into various shapes including microspheres and fibers (Loozen et al., 2013; Perez et al., 2014; Kim Y. B. and Kim G. H., 2015; Kim Y. B. et al., 2016; Raja and Yun, 2016). However, quick loss of mechanical strength of alginate during *in-vitro* culture and poor controllability over its internal microstructure (because of its excessive hydrophilic nature) are problematic for BTE (Kim Y. B. and Kim G. H., 2015). It has been found that alginate loses its mechanical strength by about 40% after 9 days of *in-vitro* culture (Shoichet et al., 1996). In addition, alginate has limited bioactivity due to lack of cell-binding sites, which are crucial for cell adhesion. This poor bioactivity of alginate has led researchers to modify it with cell-adhesive ligands or combine it with other biomaterials to promote cell responses in bone scaffolds (Di Giuseppe et al., 2018; Luo et al., 2018; Sarker et al., 2019). Bioactivity of alginate has been improved by modifying its surface using arginine-glycine-aspartate peptide coating (Genes et al., 2004). In addition, combining alginate with other hydrogels such as chitosan and gelatin has also created an suitable environment for bioprinting of bone-marrow derived mesenchymal stem cells (BMSCs) (Huang et al., 2016) and mesenchymal dental pulp derived stem cells (DPSCs) (Park et al., 2015). Further, alginate combined with Matrigel, which is a gelatinous protein mixture, has been used for encapsulation of endothelial progenitor cells (EPCs) (Poldervaart et al., 2014).

**TABLE 1:** Benefits and potential limitations of natural hydrogels commonly used in bioprinted bone scaffolds.

Hydrogel	Benefits	Potential limitations	References
Alginate	• Low price • Easy to fabricate 3D structures • Good biocompatibility • Easy gelation • High biodegradability and low immunological stimulation • Retains cell viability and osmolar requirements of cells • A suitable material for 3D printing due to crosslinkable features • Shear-thinning behavior	• Bioinert • Limited long-term stability • Rapid loss of mechanical properties during *in-vitro* culture • Limited 3D shape-ability	(Neufurth et al., 2014; Chimene et al., 2016; Narayanan et al., 2016; You et al., 2016; Luo et al., 2017; Park et al., 2017; Aldaadaa et al., 2018; Di Giuseppe et al., 2018; Milazzo et al., 2019)
Gelatin	• Accelerates gelation time • Capable of reversible thermal gelation • Biocompatible • Biodegradable	• Poor mechanical properties • High degradation rate	(Luo et al., 2015, 2018)
Chitosan	• Ingredients resemble ECM components of native tissue • Non-toxic by-products • Induces cell adhesion proliferation, differentiation, and viability	• Slow gelation rate • Poor mechanical properties • Can conflict with printing of cells and pH-sensitive molecules	(Suh and Matthew, 2000; Muzzarelli et al., 2012; Demirtaş et al., 2017)
Collagen	• Low immunogenicity • Good biocompatibility • Biodegradability • Regulates cell adhesion and differentiation	• Poor mechanical properties • Loses shape and consistency • Low viscosity and slow gelation	(Rhee et al., 2016; Mohseni et al., 2018; Turnbull et al., 2018)
Hyaluronic acid	• Good biocompatibility • Non-toxic degradation by-products • Visco-elastic properties • Highly hydrophilic • Anti-microbial properties	• Poor mechanical strength	(Kim et al., 2007; Zhu et al., 2017; Ashammakhi et al., 2019; Zhai et al., 2020)

Gelatin is another candidate for supporting cellular functions, including cell attachment, proliferation, and differentiation given that it has cell-ligand motifs (Luo et al., 2015; Milazzo et al., 2019). In addition, the thermoresponsive behavior of gelatin has made it a popular polymer for bioinks (Milazzo et al., 2019). However, gelatin has a high degradation rate and poor mechanical strength, which can be problematic for BTE (Gautam et al., 2014). Therefore, gelatin in combination with other biomaterials, such as chitosan, alginate, fibrinogen, HA, and silk fibroin, has been used as a cell carrier in bioprinting systems for BTE applications (Das et al., 2015; Huang et al., 2016; Kang et al., 2016). Methacrylamide gelatin (MG), a modified form of gelatin, has also exhibited excellent potential for cell printing with high cell viability (>97%) (Billiet et al., 2014), indicating MG can be a good synthetic hydrogel for BTE. Additionally, MG has been used as a suitable matrix for bioprinting BMSCs and the growth factor bone morphogenetic protein-2 (BMP-2) (Du et al., 2015). Coating titanium implants using gelatin methacryloyl (GelMA), another modified form of gelatin, has also improved the osteointegration of titanium implants (McBeth et al., 2017), indicating that GelMA is a good bioink candidate for BTE as well. Crosslinking strategies has also been implemented to stabilize and improve the mechanical properties of gelatin. In this regard, chemicals such as 1-ethyl-3-(3-dimethylaminopropyl) carbodiimide (EDC) (Huh et al., 2018), glutaraldehyde (Martínez-Vázquez et al., 2015), or genipin (Akkineni et al., 2016) have been applied.

Chitosan is also a favorable biomaterial for biomedical applications. This cationic polymer has a hydrophilic surface improving cell adhesion, proliferation, differentiation, and viability. The high charge density of chitosan in solution helps chitosan form insoluble ionic complexes with water-soluble anionic polymers such as alginate (Suh and Matthew, 2000; Zhu et al., 2011; Muzzarelli et al., 2012; Bakhsheshi-Rad et al., 2019). Chitosan has shown good potential as a carrier for rabbit BMSCs and the growth factor BMP-2 in hybrid polycaprolactone (PCL)/chitosan scaffolds. This combination has made a biomimetic micro-environment with improved cell retention, growth, and distribution (Dong et al., 2017).

Collagen provides excellent characteristics such as low immunogenicity, permeability, good biocompatibility and biodegradability, and has potential to regulate the morphology, adhesion, migration, and differentiation of cells (Mohseni et al., 2018; Turnbull et al., 2018). Collagen type I is the most commonly used form of collagen in bioprinted bone scaffolds (Semba et al., 2020). Collagen type I incorporation provided sufficient adhesion ligands in alginate-polyvinyl alcohol (PVA)-HAp for attachment and proliferation of pre-osteoblast cells (MC3T3-E1) (Bendtsen et al., 2017). Coating of scaffolds with collagen has also improved adhesion, proliferation, and osteogenic differentiation of osteoblast-like cells (MG63) (Vandrovcová et al., 2011) and osteogenic differentiation of human adipose-derived stem cells (hADSCs) (Linh et al., 2020). Collagen type I is not highly favorable for bioprinting bone scaffolds due to its slow gelation kinetics and low viscosity (Ashammakhi et al., 2019). A bioprintable form of collagen type I, however, has been made by adding agarose to its matrix, resulting in printed mesenchymal stem cells (MSCs) (Duarte Campos et al., 2016). Also, combining collagen type I with HA has resulted in osteochondral scaffolds with a well-suited ECM for osteoblasts (Park et al., 2014).

HA is a hydrophilic natural polymer which is well-known to be a major component of ECM in connective tissues of all mammals (Bae et al., 2011). HA has potential to be used in biomedical applications due to its biocompatibility, non-toxic degradation by-products, visco-elastic properties, and capability to retain water which keeps tissues hydrated (Bae et al., 2011; Zhu et al., 2017; Ashammakhi et al., 2019). Further, anti-microbial properties of HA makes it a good candidate for implantation in bone defects (Zhai et al., 2020). However, the natural form of HA hydrogel is easily degraded in water due to its weak mechanical strength. To overcome this limitation, a modified form of the HA network, such as acrylated HA followed by ultraviolet (UV) light crosslinking, has been used to make a suitable matrix with tunable mechanical and degradation properties for cell and growth factor encapsulation in BTE (Kim et al., 2007). Photo-crosslinked methacrylated hyaluronic acid (MeHA), another chemically-modified form of HA, also appears to be a good matrix for bioprinting in BTE as *in-vitro* research indicated good levels of cell viability (64.4%) and osteogenic differentiation (Poldervaart et al., 2017). Also, HA in combination with methylcellulose has also exhibited good capability for bioprinting of MSCs (Law et al., 2018).

The majority of natural hydrogels share a common drawback related to insufficient mechanical properties which do not mimic native bone (Baino et al., 2015). To address this limitation, enhanced mechanical performance of bioprinted scaffolds has been pursued using synthetic polymers (Gao et al., 2014). For instance, bioprinted bone scaffolds made of poly (ethylene glycol) dimethacrylate (PEGDMA) and HAp exhibited an elastic modulus of ∼359 kPa, which did not meet that of natural bone but was higher relative to natural polymers (less than 5 kPa) (Gao et al., 2014). Poly (lactic-co-glycolic acid) (PLGA) and poly (ethylene glycol) (PEG) blend has been another successful synthetic polymer for bioprinting of mechanically strong constructs (E∼57 MPa) (Sawkins et al., 2015) consisting of immortalized human mesenchymal stem cells (hMSCs) for bone repair.

#### Cells

Cells selected for BTE applications should mimic the physiological state of native cells and be able to maintain their function *in-vivo*. Also, cell proliferation under both *in-vitro* and *in-vivo* conditions must occur in a controlled manner. An implanted scaffold may fail because of too little proliferation. On the other hand, excessive proliferation may cause a lack of enough oxygen and nutrient delivery to all cells and consequently, cell apoptosis occurs. Further, the timing of cell proliferation is also of great importance such that an initial high cell proliferation be desirable but it must be sustained at a specific rate (Murphy and Atala, 2014).

Various types of cells, including stem cells and cell lines, have been utilized in bioprinting of bone scaffolds. Osteoblast cell lines are used for bone repair and regeneration in clinical applications because they are bone-forming cells engaged in formation and mineralization of bone matrix. Restricted *in-vitro* proliferation is the major drawback which is associated with usage of fully differentiated osteoblasts (Kargozar et al., 2019). The most common cell line encapsulated in bone scaffolds by means of bioprinting has been MC3T3-E1 pre-osteoblast cells which have shown the promising ability to differentiate into mature osteoblasts (Raja and Yun, 2016; Bendtsen et al., 2017; Demirtaş et al., 2017). In addition, the MG63 cell line has been utilized in 3D scaffold-based osteosarcoma models to improve tumor therapy outcomes (Bassi et al., 2020) and in 3D printed scaffolds to study bone regeneration (McBeth et al., 2017). Related to bioprinted bone scaffolds, MG63 cells were also successfully laden into 3D printed scaffolds of PCL/alginate and the *in-vitro* osteogenic activity of cell-laden scaffolds was found to be superior compared to non cell-laden scaffolds (Kim Y. B. et al., 2016). Human osteogenic sarcoma cells (SaOS-2) are another category of bone-related cells that have been employed in bioprinted bone scaffolds (Neufurth et al., 2014).

Undifferentiated stem cells (*e.g.*, MSCs), which can be isolated from a number of tissues including bone marrow and adipose tissue, have also been extensively used in biomedical applications (Su et al., 2012; Qi et al., 2016) (it is important to note that undifferentiated stem cells and cell lines need to be cultured in a medium which is supplemented with ascorbic acid, dexamethasone, and -glycerophosphate for osteogenic activity (Kargozar et al., 2019)). Stem cells have been introduced as a suitable cell source in BTE owing to their distinct characteristics, including self-renewal and good capability to differentiate into various cell lineages (Kargozar et al., 2019). MSCs are easily expanded *in-vitro* and can proliferate and differentiate into cell lineages such as osteoblasts, chondrocytes, and adipocytes. Stem cells such as BMSCs harvested from Sprague-Dawley rats (Du et al., 2015; Huang et al., 2016) and humans (Gao et al., 2014; Poldervaart et al., 2017), hADSCs (Murphy et al., 2017), as well as human amniotic fluid-derived stem cells (hAFSCs), have been used in bioprinting of bone scaffolds. The main reasons for using ASCs in bioprinted bone scaffolds are ease of availability and great proliferation rate (Kargozar et al., 2019). Although the number of cells harvested from 1 g of adipose tissue is about 500 times greater than that harvested from the same amount of bone marrow (Kargozar et al., 2019), ASCs have been found to have lower osteogenic activity compared to BMSCs (Kargozar et al., 2019). The main disadvantages of BMSCs, however, are related to the culturing process and isolation, both of which are time-consuming (Kargozar et al., 2019). DPSCs are another type of stem cells that have been successfully encapsulated in bone scaffolds by bioprinting. Compared to MSCs derived from bone marrow, DPSCs have greater potential for osteogenic differentiation and induction of vasculature (Park et al., 2015). Human nasal inferior turbinate tissue-derived mesenchymal stromal cells (hTMSCs) are also another promising source of cells that have been used in bioprinted bone scaffolds (Das et al., 2015). The hTMSCs express a proliferation rate five times higher than that of BMSCs and display approximately 30 times higher yield than that of ADSCs (Das et al., 2015). Also, parameters such as the passage number and donor age do not significantly impact the differentiation characteristics of hTMSCs (Pati et al., 2015). Muscle-derived stem/stromal cells, which are a population of self-renewing cells, has been another source of cells used in bioprinted bone scaffolds (Phillippi et al., 2008). Human umbilical vein endothelial cells (HUVECs) have also been used in bioprinting of bone scaffolds given they contribute to osteogenesis via secretion of regulatory molecules such as growth factors (Chen et al., 2018).

Taken together, this review indicates that there is no simple process for identifying one type of cell best suited for BTE. The selection criteria for cell type depends on factors such as availability, ease of isolation and culturing as well as cost of treatment (Kumar et al., 2019). Parameters such as the type of biomaterial and bioprinting technique also need to be considered regarding cell type selection.

#### Growth Factors

For treating large bone defects, vascularization still remains a challenge for BTE. One strategy used to enhance vascularization in bioprinted bone scaffolds has been usage of growth factors (Shahabipour et al., 2020). Biological aids, such as growth factors, play an important role in providing signals at damaged sites, which enable cells to migrate and stimulate the healing process (Salgado et al., 2004; Bishop et al., 2017). Accordingly, new bone formation is regulated by a range of growth factors and biomolecules, which can be included as a component of the bioink or added to the printed scaffold (Mouriño and Boccaccini, 2009; Turnbull et al., 2018).

The most common growth factor used in BTE has been the osteoinductive BMP-2 (Phillippi et al., 2008; Danhier et al., 2012; Jun et al., 2013; Poldervaart et al., 2013; Du et al., 2015). BMPs recruit MSCs to the healing location and differentiate them into the osteogenic lineage. The mechanism by which they do this is not fully understood; however, it is known that BMP-2 is the most effective inducer of osteoblastic differentiation (Salgado et al., 2004; Bai et al., 2013; Wang and Yeung, 2017). To stimulate osteogenesis, the concentration of BMP-2 has been found to be important and is dependent on the animal model. It has been reported that 0.2–0.4 mg/ml of BMP-2 is favorably osteoinductive in rats while higher concentrations of 0.43 mg/ml and 0.75–1.5 mg/ml are required for sheep and primates, respectively. However, concentrations above 1.5 mg/ml for BMP-2 have been found to cause toxic side effects (Bao et al., 2017).

Vascular endothelial growth factor (VEGF), which is found in a variety of vascularized tissues including bone, is commonly used in bone scaffolds due to its role to induce angiogenesis. VEGF regulates vascularization by recruitment of endothelial cells and play an important role to improve bone healing through both intramembranous and endochondral ossification (Salgado et al., 2004; Bai et al., 2013; Park et al., 2015; Stegen et al., 2015; Fahimipour et al., 2017; Shahabipour et al., 2020). Like BMP-2, the dosage of VEGF used in a bone scaffold should be taken into consideration. High concentrations of VEGF may cause toxicity, as well as non-functional vasculature (Dreyer et al., 2020). A concentration of 2.6 μg/animal has been found to be the highest allowable dosage of VEGF (Dreyer et al., 2020).

Compared to the effect of structures containing either BMP-2 or VEGF alone, dual delivery better promotes bone regeneration (Patel et al., 2008; Park et al., 2015). For example, dual delivery of BMP-2 and VEGF in bioprinted scaffolds of gelatin/alginate resulted in enhanced vascularization, which, in turn, promoted bone formation (Park et al., 2015). Most importantly, the specific ratio of BMP-2 and VEGF has been found to affect the synergistic effect of their combined use (Bao et al., 2017). Inappropriate proportions of BMP-2 to VEGF can have a negative influence on repair of a CSD. The ratio of 1 BMP-2 to 4 VEGF causes inhibition on bone formation; however, the ratio of 5 to 4 and above contributes to enhancing bone formation (Bao et al., 2017).

Due to restrictions, including the rapid degradation of growth factors and deactivation by enzymes *in-vivo*, polymeric delivery systems have been widely used as carriers to maintain biological functionality as well as the sustained and controlled delivery of growth factors (Yu et al., 2008; Jun et al., 2013; Izadifar et al., 2014). PLGA is one of the most successfully used biodegradable polymers in delivery systems because its hydrolysis contributes to metabolite monomers, lactic acid and glycolic acid, which are easily metabolized by the body via the Krebs cycle. Minimal systemic toxicity is also found with PLGA for biomedical applications (Danhier et al., 2012; Izadifar M. et al., 2015). Biodegradable PLGA has been investigated in various forms including microparticles and nanoparticles for BMP-2 and VEGF delivery (Kempen et al., 2009; Yilgor et al., 2009; Lee et al., 2011; Sawkins et al., 2015; Fahimipour et al., 2017).

Gelatin microparticles have also been used as delivery systems for growth factors, both *in-vitro* and *in-vivo*, due to their biodegradability and non-toxicity characteristics (Poldervaart et al., 2013; Poldervaart et al., 2014). Gelatin microparticles act as a suitable carrier for VEGF in bioprinted alginate/Matrigel scaffolds (Poldervaart et al., 2014). Controlled release of VEGF from gelatin microparticles led to a marked increase in vascularization, *in-vivo,* when compared to scaffolds with no VEGF or VEGF-loaded scaffolds with no gelatin microparticles (fast release) (Poldervaart et al., 2014).

Also, injectable thermoresponsive hydrogels have provided suitable matrices for delivery of BMP and VEGF. Such hydrogel systems are at a solution state at room temperature and convert to a gel state at body temperature. This characteristic enables delicate substances such as cells and growth factors to be readily encapsulated into the solution by mixing and then injected to the target site in the body. For instance, injectable hydrogel systems composed of PLGA-PEG-PLGA showed good capability to encapsulate BMP-2 and VEGF and release in a sustained manner (Bao et al., 2017).

### Bioprinting Scaffolds for Bone Tissue Engineering

#### Fabrication of Bioprinted Scaffolds

**FIGURE 3:**
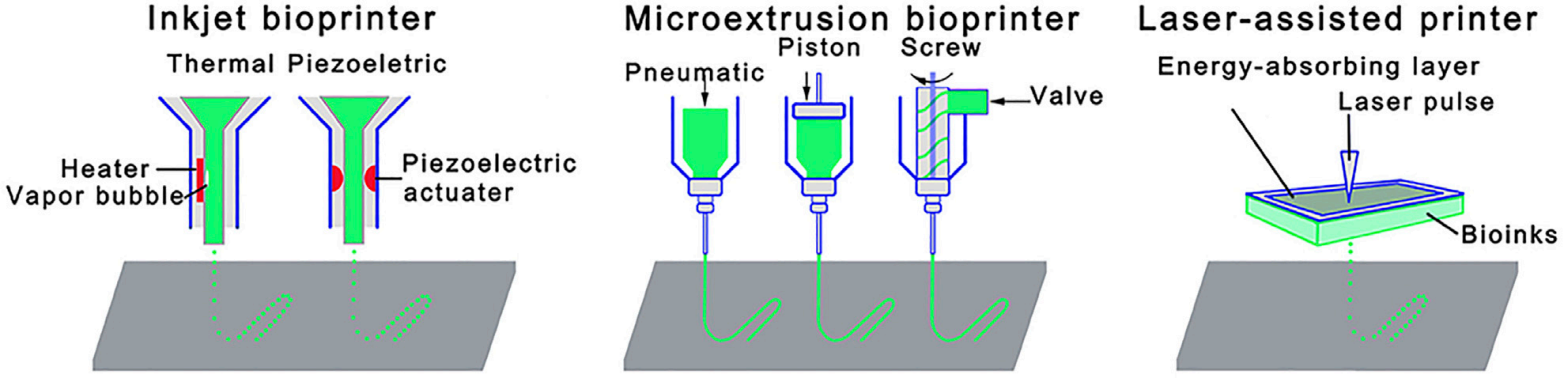
Schematic illustrations of 3D bioprinting techniques: Inkjet bioprinter; microextrusion bioprinter; and laser-assisted bioprinter. Reprinted from Li et al., (2021). Copyright 2021, Front. Bioeng. Biotechnol (Li et al., 2021).

To date, several bioprinting techniques have been used for fabricating bioprinted scaffolds with inkjet, laser-assisted, and microextrusion techniques being the three major approaches applied (Figure 3) (Li et al., 2021).

With an inkjet bioprinter, thermal or piezoelectric means are used as the driving force to print small droplets of the bioink via the nozzle (Groll et al., 2018; Ashammakhi et al., 2019). The strengths of thermal inkjet bioprinting are high printing speed and low operating costs. Although it has been reported that heat and mechanical stress involved in this technique can damage cells (Ashammakhi et al., 2019; Li et al., 2021), the technique can result in high cell viability rates given that the bioink is heated for a very short time (*e.g.*, less than 2 μs) (Gao et al., 2015). For example, hMSCs-laden peptide-conjugated PEG bone scaffolds fabricated by inkjet bioprinting showed high cell viability (∼87.9%) (Table 2) (Gao et al., 2015). Similarly, cell viability of 86.62% was shown in hMSCs-laden scaffolds of PEGDMA consisting of bioactive glass (BG) and HAp nanoparticles (nHAp), which were bioprinted by the thermal inkjet technique (Gao et al., 2014). In another BTE study (Duarte Campos et al., 2016), the viability of MSCs in various collagen I/agarose hydrogels bioprinted by the thermal inkjet technique was around 98% after 21 days of culture. This technique has been found to be safe for delicate substances as well, such as growth factors (Phillippi et al., 2008). One research group used inkjet printing to engineer stem cell microenvironments to create spatially defined patterns of immobilized BMP-2 (Phillippi et al., 2008). Thermal inkjet bioprinters, however, lack precision regarding droplet size and shape. They have also limitations regarding usage of biomaterials that are not heat or mechanically resistant. In this regard, piezoelectric-driven inkjet bioprinters can be used to overcome the limitations associated with thermal inkjet bioprinters; however, concerns of potential cell damage at 15-25 kHZ frequencies specific to piezoelectric-driven inkjet bioprinters have been mentioned (Li et al., 2021).

**TABLE 2:** Bioprinted bone scaffolds fabricated with various bioprinting techniques.

Bioink	Bioprinting technique	Cell	Ref
Alginate+HAp+PVA+Collagen	Microextrusion (The HyRel System 30 3D printer with a modified EMO-25 extruder)	MC3T3-E1	Bendtsen and Wei, (2017)
Alginate+HAp+PVA	Microextrusion (The HyRel System 30 3D printer with a modified EMO-25 extruder)	MC3T3-E1	Bendtsen et al. (2017)
Alginate, chitosan, Alginate+HAp, Chitosan+HAp	Microextrusion (The Fab@Home™)	MC3T3-E1	Demirtaş et al. (2017)
Alginate	Microextrusion (pneumatic-based)	MC3T3-E1	Raja and Yun, (2016)
Alginate+gelatin+carboxymethyl chitosan	Microextrusion (pneumatic-based)	BMSCs	Huang et al. (2016)
MG hydrogel	Microextrusion (screw-based)	BMSCs	Du et al. (2015)
Matrigel+alginate	Microextrusion (pneumatic-based)	EPCs	Poldervaart et al. (2014)
Matrigel	Microextrusion (pneumatic-based)	ASCs	Murphy et al. (2017)
Gelatin, silk fibroin	Microextrusion (pneumatic-based)	hTMSCs	Das et al. (2015)
Alginate	Microextrusion (pneumatic-based)	MG63	Kim et al. (2016b)
Alginate+gelatin, collagen	Microextrusion (pneumatic-based)	DPSCs	Park et al. (2015)
Alginate	Microextrusion (pneumatic-based)	MC3T3-E1	Ahn et al. (2013)
Alginate/gelatin, MSCs	Microextrusion (pneumatic-based) used for HUVEC-laden alginate/gelatin, a piezoelectric nozzle used for MSCs	HUVECs MSCs	Chen et al. (2018)
Acrylated PEG, acrylated peptide	Inkjet (thermal-based)	hMSCs	Gao et al. (2015)
PEGDMA with nHAp and BGs	Inkjet (thermal-based)	hMSCs	Gao et al. (2014)
collagen type I/agaros**e**	Inkjet (thermal-based)	MSCs	Duarte Campos et al. (2016)
Collagen/nHAp	LAB	Mesenchymal stromal cells	Keriquel et al. (2017)
Human osteoprogenitor cells, nHAp	LAB	Human osteoprogenitors	Catros et al. (2011)
Alginate, gelatin	Microextrusion (pneumatic-based)	SaOS-2	Neufurth et al. (2014)
Alginate, gelatin	Microextrusion (pneumatic-based)	Human endothelial cells	Akkineni et al. (2016)

Laser-assisted bioprinting (LAB), which is a nozzle-free technique, consists of three main components: 1) a laser source, 2) a ribbon coated with an absorbing layer (*e.g.*, gold or titanium), containing the bioink, and 3) a collector lying beyond the ribbon (Bishop et al., 2017). This technique starts with suspending the bioink on the bottom of the ribbon followed by evaporation induced by a laser beam, which scans over the ribbon. Afterwards, vapor bubbles propel discrete droplets onto the collector due to high pressure (Bishop et al., 2017; Li et al., 2021). LAB has advantages including printing high cell densities, high cell viability, high speed, and high degree of printing resolution (Li et al., 2021). LAB is a promising technology providing excellent control over the cell density down to the single cell level, which allows control over the functionality of cell (Keriquel et al., 2017). Successful *in-situ* bioprinting of mesenchymal stromal cells encapsulated in collagen/nHAp matrix onto a mouse calvarial bone defect has been recently performed using LAB (Table 2) (Keriquel et al., 2017). It has also been shown that LAB is an effective technique to fabricate bioprinted scaffolds made of nHAp and osteoblastic cells (Catros et al., 2011) with no change to the physico-chemical properties of nHAp nor the viability, proliferation, and phenotype of osteoblastic cells up to 15 days (Catros et al., 2011). Although it seems LAB is a promising technology for constructing tissues, it has been only used in a limited number of BTE studies. This could be due to shortcomings associated with this technique, such as the time-consuming process of ribbon preparation, potential metallic residuals in the final scaffold, and the high production cost (Mandrycky et al., 2016; Bishop et al., 2017).

In microextrusion, the bioink is loaded in a cartridge and extruded on a platform either by pneumatic or mechanical forces (*e.g.*, screw- or piston-based) (Ashammakhi et al., 2019; Naghieh and Chen, 2021). Microextrusion is the most widely used method to fabricate bioprinted bone scaffolds due to the benefits of being capable of printing a wide spectrum of biomaterials (*e.g.*, soft hydrogels, synthetic polymers, and polymer/ceramic composites) (Mandrycky et al., 2016) and high cell deposition densities (Bishop et al., 2017; Li et al., 2021). This technique can print biomaterials with a viscosity range from 30 mPa.s to over 6 × 10^7^ mPa.s (Mandrycky et al., 2016). It is able to employ either multiple nozzles to print biomaterials separately (Izadifar Z. et al., 2015; Ashammakhi et al., 2019) or co-axial nozzles to print biomaterials simultaneously such as core/shell designs (Raja and Yun, 2016). Microextrusion has been widely used to bioprint hydrogels (Table 2) such as alginate (Ahn et al., 2013; Park et al., 2015; Kim Y. B. et al., 2016; Huang et al., 2016; Raja and Yun, 2016; Demirtaş et al., 2017), chitosan (Demirtaş et al., 2017), gelatin (Park et al., 2015; Huang et al., 2016), collagen (Park et al., 2015), carboxylmethyl chitosan (Huang et al., 2016), MG (Du et al., 2015), MeHA (Poldervaart et al., 2017), and cell-laden composite scaffolds (Bendtsen et al., 2017; Bendtsen and Wei, 2017). It has been found that a wide range of cell types such as MC3T3-E1 (Bendtsen et al., 2017; Demirtaş et al., 2017), MG-63 (Kim Y. B. et al., 2016), BMSC (Du et al., 2015), DPSC (Park et al., 2015), hAFSCs (Kang et al., 2016), and hTMSCs (Du et al., 2015) can be successfully bioprinted using this technique. Though, the resolution of microextrusion bioprinters is moderate at between 50 and 500 μm (Mandrycky et al., 2016; Ashammakhi et al., 2019). In addition, cell structure and cell viability can be affected by shear stress during the printing process. However, approaches including reducing extrusion pressure or increasing the needle size can manage the cell viability issue (Bishop et al., 2017; Li et al., 2021). A comparison between bioprinting techniques including inkjet, LAB and microextrusion is given in Table 3.

**TABLE 3:** Comparison between inkjet, laser-assisted, and microextrusion bioprinting techniques.

	Inkjet	LAB	Microextrusion	Ref
Cell viability	High (>85%)	High (>95%)	Low to moderate (40–80%)	(Gao et al., 2014, 2015; Duarte Campos et al., 2016; Mandrycky et al., 2016)
Supported viscosity	Low viscosities (3.5–12 mPa.s)	Low to moderate viscosities (1–300 mPa.s)	Wide range of viscosities (30 mPa.s to over 6 × 10^7^ mPa.s)	(Chang et al., 2011; Mandrycky et al., 2016)
Printing resolution	High	High	Moderate	(Mandrycky et al., 2016; Ashammakhi et al., 2019)
Strengths	• Low-cost operation • High cell viability • Fast printing	• High resolution • Fast printing • High cell viability • Precise fabrication • Possibility of *in-situ* bioprinting • Given that it is a nozzle-free technique, it can avoid cell clogging	• Prints a wide spectrum of biomaterials • Prints high cell densities	(Mandrycky et al., 2016; Keriquel et al., 2017; Chen, 2018)
Limitations	• Lack of precision regarding droplet size and shape • Biomaterials that are not heat or mechanically resistant may be comprised • Cell damage at 15–25 kHz frequencies	• Time-consuming process of ribbon preparation • Metallic residuals in the final scaffold • High production cost	• Shear stress during printing affects cell viability • Low printing speed • Moderate resolution • Low to moderate cell viability	(Mandrycky et al., 2016; Fu et al., 2021; Li et al., 2021)

#### Printability

In 3D printing, printability is an important concept requiring the bioink to be deposited in an accurate and precise manner with high spatial and temporal control such that the printed structure replicates the virtual model (Murphy and Atala, 2014; Fu et al., 2021). Preferably, the bioink should be in liquid form before being extruded from the nozzle tip (to help avoid nozzle jamming) while after printing it should experience rapid solidification/gelation to maintain its shape (He et al., 2016).

**FIGURE 4:**
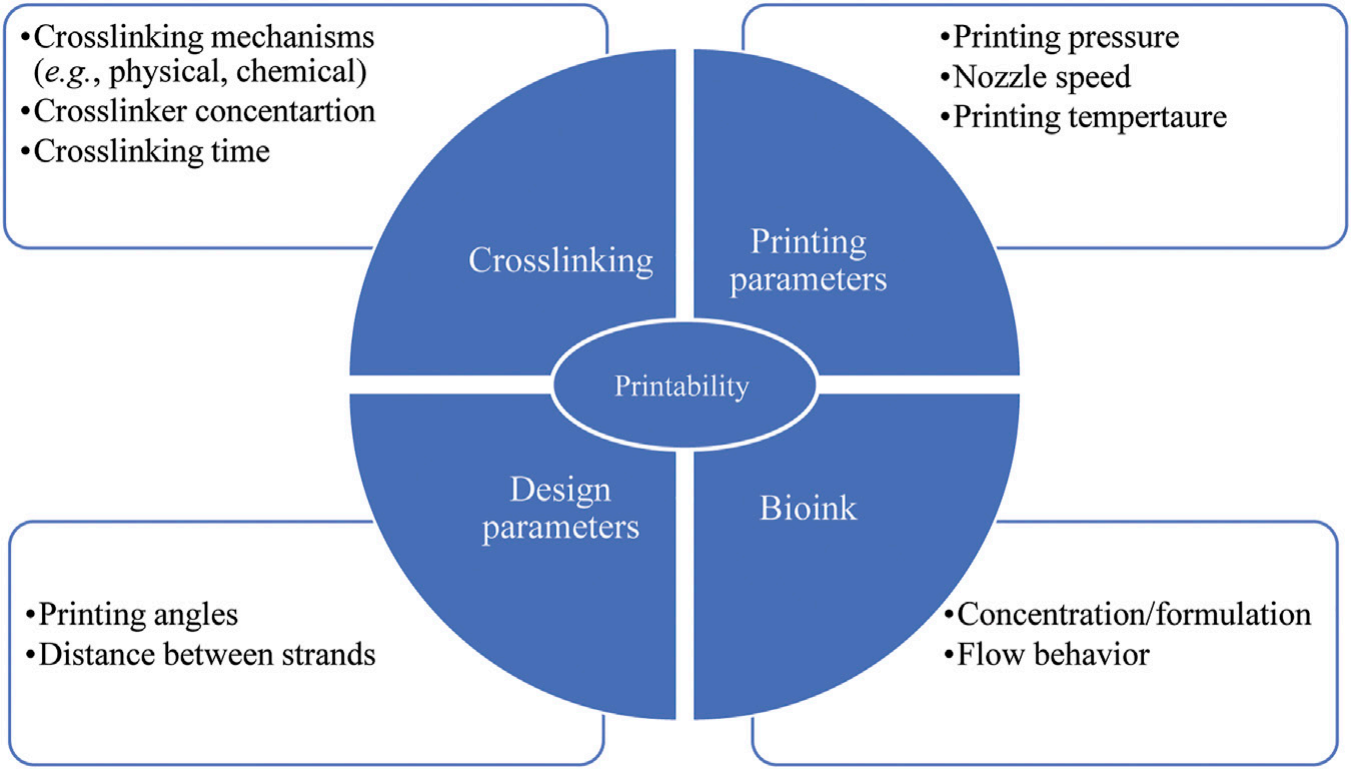
Parameters affecting scaffold printability; factors related to crosslinking, bioink and printing parameters have to be taken into consideration to achieve both favorable printability and cell viability.

There are several factors affecting bioink printability, most notably crosslinking (Figure 4) performed using either chemical or physical methods (or both) (Mandrycky et al., 2016). UV light is used as a chemical crosslinking method for GelMA hydrogel (Du et al., 2015; McBeth et al., 2017) while chemicals such as EDC/N-hydroxysuccinimide are used to crosslink collagen hydrogel chemically (Linh et al., 2020). Crosslinking can also be physical by ion gelation (*e.g.*, for alginate, chitosan, and gellan gum hydrogels) (Park et al., 2015; Akkineni et al., 2016; Kim Y. B. et al., 2016; Naghieh et al., 2018a; Sarker et al., 2018; Sadeghianmaryan et al., 2020) or by thermal gelation (*e.g.*, for collagen) (Arumugasaamy et al., 2017). When bioinks are used, crosslinker concentration must be sufficient to print structures with high printability and cell viability (Ashammakhi et al., 2019). In other words, crosslinker concentration should be sufficiently high to achieve structural integrity (*i.e.*, shape fidelity) and sufficiently low to be safe for cell function (Rajaram et al., 2014). Previous research indicates that crosslinking alginate with high concentration of CaCl_2_ (>2.5 wt%) led to cell death whereas low CaCl_2_ concentration (<2.5 wt%) contributed to a slow crosslinking rate, which in turn resulted in structures with poor shape fidelity (Raja and Yun, 2016). In another study (Kim Y. B. et al., 2016), partial crosslinking of alginate solution with a CaCl_2_/cell-laden alginate mixing ratio of 3:7 resulted in high printability as well as high cell viability (∼95%) compared to other mixing ratios (0:10, 1:9, 2:8, 4:6, 5:5).

Printability and cell viability can also be influenced by bioink concentration/formulation as well as printing temperature (Figure 4) (Ouyang et al., 2016; Sarker et al., 2019; Soltan et al., 2019; Naghieh et al., 2020). While bioinks with high viscosity provide better shape fidelity, less viscous bioinks provide a more suitable environment for cell viability due to reduced shear stress experienced during printing (Murphy and Atala, 2014; Ouyang et al., 2016). A recent study assessing the printability of gelatin/alginate bioink showed that the bioink relied on gelatin concentration. Printing the bioink with a ratio of 5% gelatin to 1% alginate at 27.5°C and 30°C resulted in fusion of subsequent layers and formation of circular interconnected channels. In contrast, printing the bioink with a ratio of 7.5% gelatin to 1% alginate at 30°C led to constructs with (preferable) distinguished layers, smooth surfaces, and square interconnected channels with regular edges. Further, printing the bioink with a ratio of 10% gelatin to 1% alginate at 25°C made constructs with irregular strands and interconnected channels. Also, high viability of embryonic stem cells was observed with gelatin/alginate bioinks printed using lower gelatin concentrations and high printing temperatures. As a result, the recommended optimized concentration and temperature for high printability and cell viability was a ratio of 7.5% gelatin to 1% alginate and 30°C, respectively (Ouyang et al., 2016). Bioink concentration/formulation also affected shape fidelity of scaffolds consisting of agarose and collagen type I (Duarte Campos et al., 2016). Here pure collagen type I was unable to form hollow structures with proper shape as complete gelation was not achieved. Increasing the agarose content in the collagen type I matrix enhanced the viscosity, gelation temperature, and consequently the accuracy of printability. Also, 98% of cell viability in various agarose/collagen type I hydrogels was observed, indicating effectiveness various hydrogel formulations and bioprinting processes for cell functionality. Further research (Raja and Yun, 2016) has shown that increasing alginate concentration from 6 wt% to 9 wt% in α-tricalcium phosphate (TCP) core/cell-laden alginate shell scaffolds resulted in high printability without detrimental effects on the viability of MC3T3-E1 cells. Another group also introduced PVA/HAp suspension into alginate formulation to improve printability without toxic effects on the viability of MC3T3-E1 cells (Bendtsen et al., 2017).

Printability can also be affected by printing pressure, nozzle speed, printing angles, and the distance between strands (Figure 4) (He et al., 2016). Overly high pressure results in quick extrusion or jetting while low pressure results in nozzle clogging. Pressure, which directly affects the flow rate, must be properly tuned to overcome the resistance of flow in the nozzle for proper extrudability (He et al., 2016; Naghieh and Chen, 2021). An overly slow or fast (translating) nozzle speed, relative to the extrusion rate, can also affect printability. Here, strands larger than the needle diameter have been created when the extrusion rate is greater than the nozzle speed, and vice versa (He et al., 2016; Gerdes et al., 2020; Fu et al., 2021). The angle of printing is also of importance as acute angles less than 90^o^ offer poor printability relative to 90^o^ right angles and obtuse angles greater than 90^o^ (He et al., 2016). Specifically, an acute printing angle leads to an overlap problem where the extrusion of hydrogel was doubled. Strand fusion is another issue arising if the distance between strands is too small (He et al., 2016). Fusion between successive layers also affects printability since it changes scaffold height, which can be problematic if a specific size is needed (He et al., 2016).

Additionally, the flow behavior of bioink has a critical influence on printability (Figure 4). From rheological point of view, a bioink should exhibit non-Newtonian shear thinning behaviour during printing with rapid viscosity recovery afterwards. With such properties viscosity is directly proportional to the applied stress, thereby allowing the bioink to be easily extruded from a nozzle tip under low pressure (Kyle et al., 2017). Such properties are also a benefit for encapsulated cells (Wu et al., 2018). Recent research (Wu et al., 2018) found excellent shear thinning and immediate viscosity recovery of bioinks composed of gellan gum and poly (ethylene glycol) diacrylate (PEGDA), which enabled constructs to print with high shape fidelity. Further, high cell viability (above 87%) over a prolonged cell culture (21 days) was found, indicating that a gellan gum/PEGDA can be an appropriate bioink for fabricating biomimetic bone.

### Mechanical and Osteoconductive Properties

#### Influence of Structural Features

Structure plays a crucial role on the mechanical and osteoconductive properties of scaffolds as well as cell functions. An adequate structure will allow flow of nutrients and oxygen into the scaffold and removal of cell waste products, thereby promoting cell survival and tissue regeneration. The degree of porosity and pore size also affects the rate of degradation (Perez and Mestres, 2016). For instance, cell proliferation was found to be 14% higher in 3D bioprinted gelatin scaffolds with pores larger than 580 μm when compared to scaffolds with a 435-μm pore size (Choi et al., 2018). Viability and proliferation of hMSCs were also higher in 3D printed PCL scaffolds with large pores due to large surface area for cells to adhere and proliferate (Domingos et al., 2013). A balance though exists between pores being sufficiently large to accommodate cells, facilitate diffusion of oxygen and nutrients, as well as facilitate waste removal without negatively affecting the scaffolds ability to bear load (Loh and Choong, 2013; Perez and Mestres, 2016). For instance, when increasing pore size from 245 to 433 μm, the compressive modulus and maximum allowable stress before failure of PCL scaffolds decreased by approximately 50 and 75%, respectively (Domingos et al., 2013). Scaffolds with small pore size possess greater load carrying capacity while scaffolds with large pore sizes contain less material, making scaffolds easier to deform (Domingos et al., 2013; Rotbaum et al., 2019). Many studies have been performed to identify the optimum pore size range for *in-vitro* cell research and *in-vivo* bone regeneration studies. Pore sizes larger than 300 µm appear to be beneficial for new bone and capillary formation; conversely, pore sizes smaller than 100 µm may not be promising for mass transport and cell migration (Roosa et al., 2010). However, there is no consensus on the optimal pore size for a bone scaffold. For example, MC3T3-E1 cells exhibited higher proliferation rates *in-vitro* with polypropylene-based scaffolds with a pore size of 350 vs. 500 µm, with the latter size found to be too large for adhesion and proliferation of cells (Lee et al., 2010). High cellular activities with small pores is thought to be due to strands being close to one other within the same layer, resulting in a high number of contact points and consequently high cell function (Domingos et al., 2013). In contrast, a pore size of 500 µm (vs. 250 µm) was found to be more effective for osteointegration and bone formation *in-vivo* with polydopamine-laced HAp/collagen/calcium silicate scaffold (Lee et al., 2019). In another study (Lee et al., 2016), *in-vivo* evaluations of PCL/HAp scaffolds exhibited higher bone regeneration with larger pores (600 and 1000 µm) when compared to scaffolds with smaller pores (200 µm). Inconsistent findings need to be interpreted with caution as other factors, including the type of biomaterials, specific material preparation technique, scaffold fabrication method as well as the type of cells, all potentially affect the specific pore size range for a bone scaffold (Roosa et al., 2010). Accordingly, more research is needed to identify optimal structures and pore sizes for scaffolds prior to its use as a bone substitute.

Internal geometry of scaffolds (*i.e.,* the arrangement of strands throughout the scaffold) has also been found to affect cell function. For example, low cell-seeding efficiency results from poor interactions between cells and scaffold material, which can be adjusted via internal geometry (Sobral et al., 2011). Cell-seeding efficiency is lower in scaffolds with homogeneous internal geometry as the culture medium has a direct path to travel within the scaffold; conversely, cell-seeding efficiency is higher in scaffolds with gradient pore sizing (Sobral et al., 2011). This improvement is attributed to an offset between scaffold layers, which affects the flow rate of the cell medium within the scaffold, thereby contributing to higher interaction between cells (Sobral et al., 2011; Yeo et al., 2012). Specifically, offset strands result in a higher number of anchorage points, thereby providing a larger surface area for cell attachment. The result is decreased cell loss and improved seeding efficiency, differentiation, and proliferation (Park et al., 2011; Perez and Mestres, 2016). For example, interlayer strand diameter offset values of 50 and 100% in PCL/β-TCP scaffolds resulted in higher seeding efficiency of MG63 cells, cell viability, alkaline phosphatase (ALP) activity, and calcium deposition when compared to scaffolds without an offset (Yeo et al., 2012)*.* Similarly, higher MSC proliferation was observed in PCL scaffolds with an interlayer offset compared to scaffolds without interlayer offset (Yilgor et al., 2008). While an interlayer offset appears to have a beneficial influence on cell function, this could vary depending upon the cell type and biomaterials employed. For example, no difference in proliferation of MC3T3-E1 cells on PCL/PLGA scaffolds (with or without interlayer offset) was found (Lee et al., 2012). Conflicting findings have also been reported regarding the influence of internal geometry on compressive properties of scaffolds. PCL/β-TCP scaffolds with 50 and 100% interlayer offset values appear to possess a higher bending modulus (+7%) than scaffolds without an offset (Yeo et al., 2012)*.* Conversely, an experimental study on PCL/HAp (40% HAp) scaffolds with an offset exhibited lower compressive elastic moduli (−50%) relative to scaffolds without an offset (Park et al., 2011) Another PCL/HAp (5% HAp) study also found a lower compressive modulus (−40%) with scaffolds having an interlayer offset (Buyuksungur et al., 2017). Rationale for lower elastic moduli was attributed to the shifted strands being unable to support overlying strands when loaded. It is worthwhile note though that other studies of PCL/HAp (Pierantozzi et al., 2020) and PCL/PLGA scaffolds (Lee et al., 2012) found no difference in the compressive moduli of scaffolds with and without an interlayer offset. Further research is needed to identify an appropriate offset which maximizes cell function while simultaneously achieving mechanical properties mimicking native bone.

The shape and geometry (*e.g.*, rectangular, triangular) of internal pores (*i.e.*, shape of embedded pores within scaffold) is also another structural property affecting cell function, osteoconductive, and mechanical properties of a bone scaffold (Sobral et al., 2011). Specifically, pore shape and geometry can be altered using specific lay-down patterns (Domingos et al., 2013). For instance, quadrangular, triangular, and complex polygonal pores have been created using 0°/90°, 0°/60°/120°, and 0°/45°/90°/135° laydown patterns, respectively. As readily apparent, increasing the number of deposition angles (*i.e.*, smaller deposition angles) in the 3D printing process creates pores with more complex geometry. Mechanical property wise, PCL scaffolds with quadrangular pores exhibited higher compressive moduli (∼34.2 MPa) relative to triangular (∼30.5 MPa) and polygonal pores (∼19.1 MPa) (Domingos et al., 2013). Rationale was attributed to adjacent layers sliding more easily with scaffolds made using smaller deposition angles (Domingos et al., 2013). Sliding then resulted in more deformation and lower compressive moduli. Deposition angles have also affected the cell viability whereby lower viability of hMSCs was noted with small deposition angles [125]. Rationale for these findings is likely due to limited cell accessibility and colonization with smaller angles (Domingos et al., 2013). Interestingly, hexagonal-shaped pores appear to offer both high cell viability and strength. Glass-ceramic scaffolds with hexagonal-shaped pores were found to possess compressive strength (S_c_∼122 MPa) comparable to cortical bone and elastic moduli (E∼2.4 GPa) comparable to trabecular bone (Roohani-Esfahani et al., 2016). Rationale was attributed to high contact area between strands in subsequent layers as well as the creation of a highly anisotropic structure which enhanced load transfer when compared to other patterns (*e.g.*, zigzag, curved, rectangular) (Roohani-Esfahani et al., 2016). Similar findings were reported by Van Bael et al. (2012) where Ti6Al4V bone scaffolds with hexagonal pores showed higher elastic moduli (∼11.3 GPa) relative to rectangular (∼2.8 GPa) and triangular pores (∼2.04 GPa). In addition, hexagonal pores exhibited the highest cell growth, followed by rectangular and triangular pores. Rationale was due to high number of corners with hexagonal pores, which permitted rapid cell bridging as the distance between strands was shorter relative to other configurations (Van Bael et al., 2012).

#### Influence of Bioactive Ceramics

To date, bioceramics have been largely used to help repair and reconstruct diseased or damaged living tissues and organs of the body (Baino et al., 2015; Sadeghianmaryan et al., 2022). The use of bioactive ceramics in combination with polymers in BTE has gained interest as the resulting constructs possess bioactivity and high compressive strength/moduli provided by the ceramic phase while the polymeric network provides toughness, flexibility, and biodegradability (Huang et al., 2018; Kumar et al., 2019).

Mechanical properties of bioprinted scaffolds are of importance because they affect osteogenic differentiation and cell morphology (Kim et al., 2017). In cell-laden scaffolds, enhanced mechanical and osteoconductive properties have also been made using bioceramics such as TCP and HAp (Kim et al., 2017; Ashammakhi et al., 2019). TCP contains two crystals namely α-TCP and β-TCP, where α has higher solubility than β. In an aqueous medium (*e.g.*, culturing condition in minimum essential media alpha), α-TCP shows a cementic reaction which hardens the bioceramic and subsequently forms calcium-deficient hydroxyl apatite. Although two crystals of TCP have similar chemical structure, α-TCP demonstrates more rapid bone formation *in-vivo* compared to β-TCP. Mechanically, cell-laden collagen-coated α-TCP/collagen scaffolds showed lower elastic modulus (0.55 MPa) relative to that of trabecular bone but the elastic modulus was markedly higher than pure cell-laden collagen (0.04 MPa) (Kim et al., 2017). Biological activity wise, cell-laden collagen-coated α-TCP/collagen scaffolds showed higher osteoconductive properties (ALP activity, osteopontin (OPN), and calcium deposition) compared to pure collagen (Kim et al., 2017).

HAp, in particular, has been widely explored in BTE studies due to its biocompatibility, osteoconductivity as well as close compositional and mineralogical similarities to the inorganic component of natural bone (Wiria et al., 2007; Kim et al., 2012; Serra et al., 2013; Qi et al., 2016; Bendtsen et al., 2017; Wang et al., 2017; Kumar et al., 2019). With regards to mechanics, HAp exhibits an elastic modulus of 35–120 GPa and a compressive strength of 120–900 MPa (Milazzo et al., 2019). Related higher MC3T3-E1 cell viability (∼96%) was observed with an optimized formulation of bioprinted PVA/HAp/alginate, attributed to the incorporation of PVA/HAp compared to that of cell-laden alginate scaffolds (∼60%) (Bendtsen et al., 2017). Various formulations of PVA/HAp/alginate scaffolds encapsulated with MC3T3-E1 cells showed an elastic modulus (∼2–10 kPa) lower than that of trabecular bone, but the scaffolds remained intact over 14 days incubation in culture media. These results suggest that cell-laden PVA/HAp/alginate scaffolds could support cellular activity for 14 days *in-vitro* (Bendtsen et al., 2017). Research on hASCs-laden alginate/gelatin/nHAp also showed that incorporation of nHAp particles improved osteogenic activity and bone formation both *in-vitro* (osteogenesis-related genes) and *in-vivo* (subcutaneously implanted) when compared to pure hydrogel (Wang et al., 2016). Additionally, adding HAp into bioprinted hASCs-loaded hydrogels (gelatin methacrylate/HA) supported bone matrix mineralization, as confirmed by biomarkers (including collagen type I, ALP, and OPN), making this HAp-modified hydrogel a promising bioink for bone bioprinting (Wenz et al., 2017). Adding HAp into chitosan and alginate hydrogels also increased osteogenic gene expression and enhanced bone mineral density relative to pure hydrogels (Demirtaş et al., 2017). Taken together, prior research indicates that HAp plays an important role in adhesion, growth, proliferation, and differentiation of osteogenesis-related cells. HAp incorporation into alginate formulation also provides a suitable environment for differentiation of MC3T3-E1 cells into osteoblasts as well as calcium deposition (Bendtsen and Wei, 2017; Kumar et al., 2019).

Bioactive glasses are another category of bioceramics that have received increasing attention for fabricating bone scaffolds with 3D printing technology. BGs have the capability to bond to native bone tissue, thereby providing a stable interface needed for a range of biological functions such as angiogenesis and tissue regeneration (Baino et al., 2015). When BGs are used, rapid release of ion dissolution products leads to the formation of a nHAp layer on the BG surface, which can interface with host tissue (Baino and Vitale-Brovarone, 2014; Murphy et al., 2017; Baino and Fiume, 2020). Another advantage associated with using BGs is that their chemical composition and subsequent degradation rate can be tailored. Accordingly, scaffolds containing BGs can be designed with a degradation rate matching that of bone ingrowth and remodeling (Fu et al., 2011). For example, hybrid bioprinted scaffolds consisting of PCL/BGs, along with ASCs-loaded Matrigel, showed ∼23% weight loss over 2 weeks and strong bioactivity via formation of HAp crystals (Murphy et al., 2017). However, there is limited information regarding the use of BGs in 3D bioprinted bone scaffolds, as well as its effect on vascularization and bone formation *in-vivo*.

#### Influence of Hybrid Systems

The mechanical and osteoconductive properties of bone scaffolds have also been adjusted through the fabrication of hybrid scaffolds. In a hybrid system, scaffolds are printed using two materials with different mechanical and biological properties (Akkineni et al., 2016; Raja and Yun, 2016). The stiffer material carries the majority of the applied load and thereby shields the (less stiff) softer material. Soft natural hydrogels (*e.g.*, collagen, gelatin, and alginate), can then be used for embedding delicate substances including cells or growth factors (Park et al., 2015; Akkineni et al., 2016; Kim Y. B. et al., 2016). Synthetic polymers, such as PCL, have been commonly used in hybrid scaffolds as the stiffer material. Cell-laden hybrid scaffolds of PCL/chitosan showed a compressive strength of ∼7 MPa (comparable to trabecular bone with S_c_ ≈ 2–12 MPa (Wu et al., 2014)), and much higher than that of chitosan (<1 MPa) (Dong et al., 2017). In addition, higher cell retention, proliferation, bone matrix formation, and evidence of osteogenesis (*e.g.*, via presence of collagen type I, osteocalcin (OCN), ALP) were found in cell-laden PCL/chitosan hybrid scaffolds compared to PCL scaffolds. Elastic moduli of 2–13 MPa have been observed with hybrid scaffolds containing PCL/alginate strands, with various porosities (*i.e.*, 41, 62, 66%) coated by cell-laden alginate bioink (Kim Y. B. et al., 2016). Although the resulting elastic moduli were much lower than that of native trabecular bone (E ≈ 100–5000 MPa (Wu et al., 2014)), it was higher than that of pure cell-laden alginate (E ≈ 3.6–32.1 kPa (Naghieh et al., 2018b)). A research including both *in-vitro* and *in-vivo* studies on hybrid PCL scaffolds loaded with bioprinted HAp-modified alginate suggested such hybrid system for osteochondral regeneration; however, no mechanical evaluation was done in this study (You et al., 2018).

**FIGURE 5:**
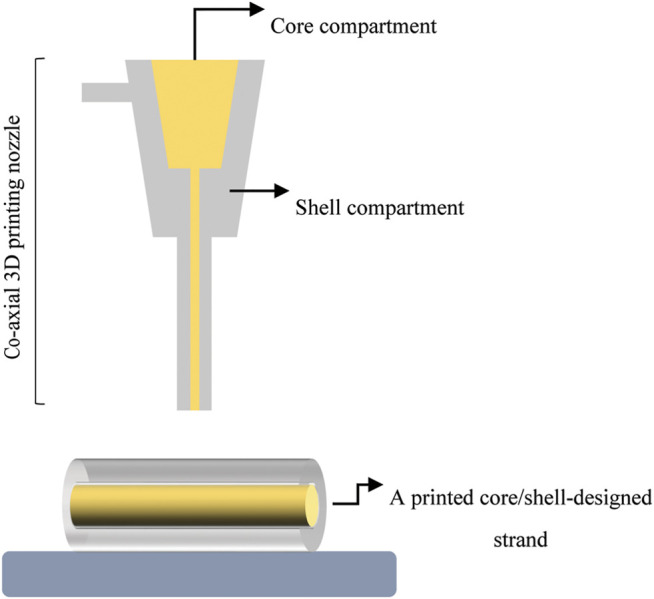
A schematic illustrating core/shell designed strand fabricated with a co-axial 3D printing nozzle.

Specialized arrangements, such as core/shell designs (Figure 5), have shown promise as new hybrid scaffolds (Perez and Kim, 2015). With such designs, the structure is made of a soft inner core surrounded by a stiff outer shell (Akkineni et al., 2016). Here the core would contain cells or growth factors (Perez et al., 2015). The core has also been reinforced with a stiffer material such as PCL (Kim M. and Kim G. H., 2015), a higher hydrogel concentration (Ahn et al., 2013), and a bioactive ceramic (Raja and Yun, 2016). Core/shell scaffold designs comprised of a collagen hydrogel (0.6 wt%) as the core and high concentration alginate hydrogel (16.7 wt%) as the shell exhibited a higher elastic modulus of ∼200 MPa compared to scaffolds with pure alginate strands in both the core and shell (E∼1 MPa) (Akkineni et al., 2016). With this approach, hybrid core/shell scaffold designs comprised of collagen/alginate reached the lower end of elastic moduli for trabecular bone (E ≈ 100–5000 MPa (Wu et al., 2014)). Also, bioprinted scaffolds consisting of a α-TCP core and cell-laden alginate shell exhibited a compressive strength of 3.2 MPa (Raja and Yun, 2016), comparable to that of trabecular bone (S_c_ ≈ 2–12 MPa (Wu et al., 2014)), while the strength of pure alginate hydrogel was less than 0.5 MPa. Biologically, MC3T3-E1 cells encapsulated in the alginate shell were able to maintain their viability (>90%) for a long culture period (35 days) (Raja and Yun, 2016). In light of core/shell scaffolds, mechanical findings showed that adding HAp into the shell formulation not only led to 1.8-fold increase in elastic modulus of core (PCL)/shell (gelatin/PVA/HAp) scaffolds, but also resulted in superior ALP activity and calcium mineralization of MG-63 cells relative to HAp-free formulation (Kim et al., 2020). Hybrid scaffolds are also highly beneficial for dual release of growth factors both temporally and spatially to induce bone regeneration effectively (Park et al., 2015; Perez et al., 2015; Akkineni et al., 2016). In this regard, a novel 3D printing system printed two different hydrogels within a PCL framework: 1) a collagen hydrogel loaded with DPSC/BMP-2 in the periphery zone; and 2) an alginate/gelatin hydrogel loaded with DPSC/VEGF in center zone. This approach enables either dual release or sequential delivery of two growth factors for vascularized BTE (Park et al., 2015).

### Tracking Bone Regeneration

#### In-Vitro

It is necessary to assess the performance of bone tissue-engineered constructs using pre-clinical *in-vitro* studies prior to evaluating therapeutic feasibility in animal studies (Salgado et al., 2004). In BTE studies, bone-specific biomarkers including enzymes (ALP) and proteins (OPN, OCN, and collagen type I) have been commonly studied to evaluate the osteogenic activity of scaffolds *in-vitro* (Li et al., 2016). ALP is an enzyme found in the bloodstream, with most of ALP produced in the liver and some within bones, intestines and kidneys (Kuo and Chen, 2017). It is an initial biomarker used to assess osteogenic differentiation and phenotype (Kim SE. et al., 2016). Additionally, OCN, which is the most abundant non-collagenous protein in bone, has been used as a biomarker of osteoblast function to assess bone formation (Kuo and Chen, 2017). OPN, which also belongs to the family of non-collagenous proteins, is secreted by several cells (such as osteoblasts and osteoclasts) and plays a crucial role in bone remodeling and biomineralization (Singh et al., 2018). Collagen type I is also the major protein component of the ECM in bone and its expression is often studied to evaluate osteogenic activity (Bao et al., 2017). Further, the expression of genes including Runt-related transcription factor-2, which is the master gene related to osteoblast differentiation, as well as osterix has also been studied with respect to pre-osteoblastic stage. The expression of these bone biomarkers is commonly identified by biochemical analysis including real time reverse transcription-polymerase chain reaction (Bao et al., 2017; Demirtaş et al., 2017; Liu et al., 2019) (Dong et al., 2017). After osteogenic differentiation stage, differentiated cells start to secrete a mineralized matrix resulting in calcium deposition, which is used as a biomarker for mature osteoblasts and is usually assessed by Alizarin Red Staining (Florencio-Silva et al., 2015; Kim Y. B. et al., 2016; Hwang and Horton, 2019; Pierantozzi et al., 2020).

Accordingly, cell viability and cell proliferation are other important parameters to be assessed. This is needed as encapsulated cells may experience high shear stress during the bioprinting process as well as excessive crosslinking (Zhang et al., 2017). The type of cells and biomaterials present may also affect the viability of cells (Ashammakhi et al., 2019). In bioprinted scaffolds, colorimetric assays including live/dead, MTT (Thiazolyl Blue Tetrazolium Bromide) or Presto Blue (PB) have been used to study cell viability and subsequently cell proliferation (Akkineni et al., 2016; Kim Y. B. et al., 2016; Demirtaş et al., 2017; Zhang et al., 2021). Recently, PB has been offered as an alternative to MTT to assess the viability and proliferation of MC3T3-E1 cells (Demirtaş et al., 2017) as well as human umbilical vein endothelial cells (Boncler et al., 2014). The major benefit of PB assay is that it is resazurin-based which is a water-soluble dye and consequently non-toxic to cells. Whereas MTT is a tetrazolium-based by which MTT converts into purple formazan compound in metabolically active cells. Formazan product must be solubilized in dimethyl sulfoxide which can cause cytotoxicity. Further, PB assay is faster due to shorter incubation time needed (10 min to 2 h) (Boncler et al., 2014) compared to that of MTT (2–4 h) (Boncler et al., 2014; Pierantozzi et al., 2020). However, the gap observed here is that PB assay has only been used for a limited number of cells in comparison with MTT and further investigation is needed.

#### In-Vivo

Although *in-vitro* studies are critical stepping stones, they cannot fully reflect *in-vivo* models which are the ultimate test for testing the efficacy of bioprinted scaffolds. Consequently, pre-clinical (animal) *in-vivo* studies are needed to study integration and function of bone scaffolds (Ashammakhi et al., 2019). *In-vivo* studies, however, are cost intensive, require strict ethical considerations, and a key limitation is variation between species. Specifically, bone composition, density, and mechanical properties in commonly used *in-vivo* animal models (*e.g.*, rats, pigs) are different from those of humans, which may lead to outcomes different from what would been seen in humans (Abubakar et al., 2016; Caddeo et al., 2017; Klüter et al., 2019). The choice of an animal model is thus a crucial step towards the success of pre-clinical *in-vivo* studies.

An appropriate animal model should mimic the clinical setting such that it is biologically comparable (as much as possible) to human physiology (Salgado et al., 2004). In BTE studies, much of the research has focused on rodent models (such as mice (Chuenjitkuntaworn et al., 2010; Poldervaart et al., 2013), and rats (Blum et al., 2003; Sawyer et al., 2009; Harada et al., 2014; Perez et al., 2015; Suenaga et al., 2015; Sukul et al., 2015; Kang et al., 2016; Qi et al., 2016; Bao et al., 2017; Song et al., 2017; Liu et al., 2019)) and rabbit models (Oh et al., 2007; Kim et al., 2012, 2014; Jun et al., 2013; Pae et al., 2019) due to economic considerations, reproducibility, and throughput. Usage of bigger animal models, such as goats (Yu et al., 2008) or sheep (Ahmed and Hamad, 2020), is rare mainly due to the high cost (Salgado et al., 2004; Spicer et al., 2012).

Anatomically, the calvarium has widely served as a model site to create bone defects and subsequently track bone regeneration. The calvarium provides a relatively large and accessible surface upon which to perform a surgical operation and for handling the specimen. This defect model also permits the creation of a reproducible defect, which can be quickly generated and does not require fixation for stabilization of the skeleton (unlike femoral defects) (Salgado et al., 2004; Spicer et al., 2012). In addition, a uniform circular defect can be created, which enables a convenient means to assess bone regeneration by radiographical and histological analyses (Salgado et al., 2004). However, a calvarial defect is not an appropriate defect model when intending to track bone formation and remodeling under biomechanical loading given that this anatomical site experiences little-to-no loading compared to long bones (Spicer et al., 2012).

In terms of physical size, a defect 8 mm in diameter is regarded as an acceptable CSD in the rat calvarium (Patel et al., 2008; Kang et al., 2016). Bilateral calvarial defects have also been created in a rat calvarium using subcritical-sized defects 5 mm in diameter. This side-by-side approach allows comparisons of control and treatments groups without variation caused by other factors (*e.g.*, activity levels). Accordingly, fewer animals are needed for the study design (Sawyer et al., 2009; Sohn et al., 2010; Zhang et al., 2014; Qi et al., 2016; Liu et al., 2019). However, there is potential for interactions between the two adjacent defects, which can affect study outcomes (Spicer et al., 2012).

Calvarial defects have also been used with rabbits. A key benefit is that multiple defects can be created in a single rabbit as they have a larger cranium than that of rats (Sohn et al., 2010; Pae et al., 2019). Though, the 8 mm diameter defect used with rats is too small for rabbits (Pae et al., 2019). A study of various-sized defects in rabbits indicated that a single CSD should be larger than 15 mm in diameter but two bilateral 11 mm diameter calvarial defects could be a suitable alternative (Sohn et al., 2010).

In addition to calvarial models, other sites including the tibia and femur have been used to track bone formation and regeneration within a CSD. Compared with calvarial models, tibial and femoral bone defect models are more suited when the bone scaffold will be used as a load-bearing bone graft. To date, tibial defects 1.2 cm (Bao et al., 2017), 1.5 cm (Wu et al., 2017), and 0.7 cm (Kim SE. et al., 2016) in length have been created in rabbits and rats. Femoral bone defects (diameter 5 mm, height 2.5 mm) have been applied with rabbits (Buyuksungur et al., 2017). Another alternative to calvarial models are ectopic models (*i.e.*, subcutaneous models) (Bao et al., 2017) where rats or mice are usually used. With subcutaneous models, scaffolds are implanted in the back of animal to observe bone scaffold degradation as well as vascularization prior tracking bone regeneration using bone defects (Salgado et al., 2004; Bao et al., 2017). To assess the bone regeneration capabilities of implanted bone scaffolds within CSDs, histological analyses using hematoxylin and eosin staining as well as Masson’s trichrome staining have been commonly used (Chuenjitkuntaworn et al., 2010; Liu et al., 2019). In addition, computerized imaging analysis such as micro-computed tomography (micro-CT) is a useful method to obtain information about bone, density, and structure as well as new bone integration with the host bone (Salgado et al., 2004). Table 4 summarizes several 3D bioprinted bone scaffolds that have been studied via *in-vitro*, *in-vivo,* or both to track bone regeneration as well as cell activities such as cell viability, proliferation, and differentiation.

**TABLE 4:** Tracking bone regeneration using bioprinted scaffolds.

Bioink	Bioprinting technique	Cell	Growth factor	Study type	Summary of findings	Ref
Matrigel, alginate, gelatin microparticles	Microextrusion (pneumatic-based)	EPCs	VEGF	*In-vitro* and *in-vivo* (subcutaneous implantation in mice)	• Controlled release of VEGF via gelatin microparticles was found. • Thanks to prolonged release of VEGF, significant increase *in-vivo* vascularization observed	Poldervaart et al. (2014)
Collagen, nHAp	LAB	Mesenchymal stromal cells	---	*In-vitro* and *in-vivo* (bilateral calvarial bone defect in mice)	• Cell-laden collagen/nHAp bioprinted in disk geometry exhibited significant increase in bone regeneration *in-vivo* compared to collagen/nHAp without cells and cell-laden collagen/nHAp printed in ring geometry. • Both bioprinted geometries showed an increase in metabolic activity from day 1 to day 8 post-printing	Keriquel et al. (2017)
Composite hydrogel (gelatin+Fibrinogen+hyaluronic acid+glycerol)	Microextrusion (pneumatic-based)	hAFSCs	---	*In-vitro* and *in-vivo* (single calvarial bone defect in rats)	• New bone was formed within the bioprinted scaffold. Whereas, fibrotic tissue ingrowth and minimal bone formation were found in the blank defect and the defect treated with cell-free scaffold, respectively. • Vascularization within the new formed bone was found in the bioprinted scaffolds. However, poor vascularization was observed in blank defect and the defect implanted with cell-free scaffold. Blood vessels in cell-free scaffolds were restricted to periphery portion. • Cell viability after bioprinting was found to be around 91%. • Osteogenic differentiation of hAFSCs in bioprinted scaffolds was confirmed via calcium deposition	Kang et al. (2016)
Alginate+gelatin, collagen	Microextrusion (pneumatic-based)	DPSCs	VEGF, BMP-2	*In-vitro* and *in-vivo* (subcutaneous implantation in mice)	• After bioprinting, cell viability rates in collagen and alginate/gelatin hydrogels were found to be 92 and 99%, respectively. • Presence of BMP-2 and VEGF in cell-laden hydrogels were effective for both vasculogenic and osteogenic differentiation. • Cell-laden hydrogels with BMP-2 showed higher calcium mineralization, ALP, and Runx-2 compared to cell-laden hydrogels without BMP-2. • Spatial and temporal release of growth factors were achieved using cell-laden hydrogels. • Bone regeneration was faster in pre-vascularized structures (cell-laden hydrogels containing both BMP-2 and VEGF) than in non-vascularized structures (cell-laden hydrogel with no growth factor, cell-laden hydrogel with BMP-2 only)	Park et al. (2015)
Alginate	Microextrusion (pneumatic-based)	MG63	---	*In-vitro*	• Cell survival rate was found to be around 93%, after bioprinting process. • Homogenous cell distribution was observed in bioprinted scaffolds which resulted in higher osteogenic differentiation (ALP) and calcium mineralization relative to non-bioprinted scaffolds. • Higher cell proliferation was found in cell-laden scaffolds compared to non-bioprinted scaffolds	Kim et al. (2016b)
Alginate, Chitosan, Alginate+HAp, Chitosan+HAp	Microextrusion (pneumatic-based)	MC3T3-E1	---	*In-vitro*	• The positive effect of HAp on osteogenic activity of cells in hydrogels was confirmed with bone biomarkers including ALP, OCN, collagen type I, and Runx-2. • Three and 9 days after bioprinting, average cell viabilities were found to be around 89–93% and 90–95%, respectively	Demirtaş et al. (2017)
Alginate, gelatin	Microextrusion (pneumatic-based)	SaOS-2	---	*In-vitro*	• Overlaying cell-laden hydrogel with calcium salt of polyphosphate in parallel with adding osteogenic supplements to the culture medium led to a remarkable increase in cell proliferation and calcium mineralization.	Neufurth et al. (2014)
Alginate, gelatin	Microextrusion (pneumatic-based)	Human endothelial cells	BMP-2, VEGF	*In-vitro*	• A dual delivery of BMP-2 and VEGF was achieved with core/shell bioprinting. • One day after bioprinting, human endothelial cells showed a cell viability of 66.2% and it increased to 84.5% after 21 days of culture	Akkineni et al. (2016)
Alginate, gelatin, MSCs	Microextrusion (pneumatic-based) used for HUVEC-laden alginate/gelatin, a piezoelectric nozzle used for MSCs	HUVECs MSCs	---	*In-vitro*	• Evaluation of angiogenic properties exhibited the capability of alginate/gelatin hydrogel to help form and maintain vascular network over 7 days of culture. • Findings showed that HUVEC-laden hydrogel had a positive effect on osteogenesis of MSCs as proved by bone biomarkers. • Hydrogel had a positive effect on cell proliferation throughout the bioprinted scaffold	Chen et al. (2018)
Acrylated PEG, acrylated peptide	Inkjet (thermal-based)	hMSCs	---	*In-vitro*	• Cell viability of 87.9% post-printing was found. • PEG-peptide scaffolds stimulated and increased osteogenic activity and calcium mineralization of cells for long time period (21 days)	Gao et al. (2015)

### Challenges and Recommendations for Future Research

Although bioprinting has received considerable attention as a promising technique to produce porous biomimetic scaffolds with controllable geometries, as this review indicated, challenges remain in fabricating scaffolds to repair CSDs. The most promising emerging techniques employ vertical or horizontal gradients of bioink along with gradient structures. These techniques are only beginning to be explored; however, they appear to be a promising strategy to develop constructs mimicking the design and composition of bone. Further, although many attempts have been performed to study the influence of structural features on mechanical and biological properties of bone scaffolds, this area has not gained much attention in bioprinted scaffolds, and it needs to be investigated. Complex-shaped pores including hexagonal, which have shown good potential to satisfy requirements of native bone mechanically and biologically, should be specifically explored in cell-laden scaffolds. Another limitation pertains to the restricted usage of BGs in bioprinting of bone scaffolds compared to other bioceramics such as HAp or TCP. More research is then needed in this regard.

Fabricating scaffolds with dynamic functionality is another challenge which has not yet been achieved with current bioprinting techniques. Four-dimensional (4D) emerging technology has created new avenues of research using smart biomaterials. Using 4D printing, self-folding tubes have been made upon immersion in cell culture media with stimuli-responsive biomaterials (Kirillova et al., 2017). The key benefit of this technique is that dynamic bioprinted scaffolds made of stimuli-responsive biomaterials will be able to change their shapes over time under different intrinsic and/or external stimuli (Wan et al., 2020). In addition, bioprinted scaffolds made of stimuli-responsive biomaterials will be able to adopt to the vascularization, which is the main obstacle in BTE, and cell behavior specific to the micro-environment of the defect area (Wan et al., 2020). Importantly, this approach avoids the need to create vascular-like networks in scaffolds to help repair bone defects. However, when optimizing a bioink made of stimuli-responsive biomaterials, printability and cell viability should also be taken into consideration which may be another challenge.

With regards to *in-vivo* studies, conventional micro-CT has been the most widely used method to track bone regeneration although the synchrotron radiation micro-CT provides benefits including images with greater quality, resolution, contrast, shorter scan time as well as non-destructive 3D visualization (Cooper et al., 2011). Synchrotron radiation micro-CT has been illustrated promising to perform *in-vivo* imaging to track tissue regeneration once scaffolds are implanted in live animals over duration of study (Izadifar et al., 2016; Duan et al., 2021). This state-of-the-art approach is missing in most BTE studies although it can provide researchers with useful information in terms of scaffold degradation rate, the way implant is integrated with the host tissue, vascularization, and bone formation over time.

Taken together, classic bone tissue engineering approaches (scaffold-based) has been unable to fabricate a bone organ model for clinical application to date. Organoid, which is an emerging technology in tissue engineering, has been introduced as a promising field of study to fabricate functional organs including human bone (Burdis and Kelly, 2021). Organoids are defined as “*in-vitro* 3D cellular clusters derived exclusively from embryonic stem cells, induced pluripotent stem cells or primary tissue, capable of self-renewal and self-organization, and exhibiting similar organ functionality as the tissue of origin*”* (Fatehullah et al., 2016)*.* According to Fatehullah et al. (2016), organoid may “rely on artificial ECM to facilitate their self-organization into structures that resemble native tissue architecture”. Most recently, organoids for woven bone (Akiva et al., 2021) and trabecular bone (Yongkuk et al., 2021) have been successfully developed. In addition to organoids, biofabrication of cell aggregates/spheroids (cell-based tissue engineering) has emerged as a promising approach for vascularized bone regeneration (Heo et al., 2019).

With regard to clinical applications, bioceramics, most typically calcium phosphates and BGs, have been used in the form of granules or powder as a bone filler and injectable formulations as bone cement (Baino et al., 2015). In addition, HAp-coated metal joint prostheses and hemispherical ceramic (alumina) acetabular cups have been used for hip arthroplasty (Rajaratnam et al., 2008; Baino et al., 2015). Alumina and HAp are used in clinical applications as they do not generate an immune response (Baino et al., 2015). Straumann^®^ BioCeramic™ and Bio-Oss^®^ are examples of commercial bone mineral substitutes (Sabetrasekh et al., 2010). It is important to note that current commercial applications employ a limited number of biomaterials (*e.g.*, bioceramics and metal grafts). Bioprinted bone scaffolds containing both cell-laden hydrogel and supportive polymers are potential future commercial products for clinical application.

## Conclusion/Summary

Although bone has the capacity for self-healing, bone grafts and substitutes are needed to repair CSDs. Due to the limitations associated with conventional therapeutic methods, considerable attention is being paid to BTE to repair bone defects and improve functionality. Biomedical scaffolds provide a temporary template for mechanical support, cell attachment, and induction of bone formation *in-vivo*. In this regard, scaffold structure is of great importance as it affects cell activities, waste removal, the transport of nutrients and molecules to the inner parts of the scaffold, as well as the mechanical integrity of the scaffold.

3D printing techniques are gaining prominence for creating scaffolds due to their capacity to create highly porous structures with interconnected pores. In addition, bioprinting allows for the creation of biomimetic scaffolds using different types of biomaterials (*e.g.*, natural, synthetic), cells (*e.g.*, cell lines and stem cells), and growth factors (*e.g.*, osteogenic and angiogenic). To date, various studies have aimed to create a scaffold with mechanical and osteoconductive properties mimicking native bone. Consensus though is lacking regarding optimal structural features of bone scaffolds, fabrication method, material preparation technique, cell type, biological aids, and implantation site. Accordingly, more research is needed.

Also, an important goal in tissue engineering applications has been the delivery of biological aids such as growth factors to the defect site in a controlled manner without loss of bioactivity, which is crucial for generation of new blood vessels (*i.e.*, vascularization). Nanoparticulate-delivery systems and microparticles have demonstrated good potential to achieve this BTE objective.

Although hydrogels provide an appropriate matrix for cell encapsulation, their mechanical properties do not match those of native bone. Therefore, a CSD cannot be treated with scaffolds made of hydrogel alone and supportive resistance, mimicking the mechanical properties of trabecular and cortical bone, is needed. Recently, state-of-the-art printing methods have emerged to fabricate biomedical scaffolds with specific designs, such as core/shell structures which enable the protection of cells and biomolecules while simultaneously providing a suitable platform for dual delivery or sequential delivery of growth factors into the surrounding environment. To fabricate bioprinted bone scaffolds, various including inkjet, LAB, and microextrusion have been used, with microextrusion being most used.

To track bone regeneration in bone scaffolds, *in-vitro* and *in-vivo* assessments have been done as preclinical evaluations. Despite many advances in this area, there are still challenges pertaining to these assessments. Specifically, *in-vitro* studies cannot completely mimic the *in-vivo* condition. *In-vivo* studies are also cost intensive, need strict ethical considerations, and the major limitation is bone variation between species.

No functional bone organ/construct has yet been fabricated using conventional tissue engineering and 3D printing approaches. Technologies including 4D printing, organoids, and cell aggregates/spheroids have emerged to pave this path, which are still in infant stages for treating large bone defects.
